# Microscopical Labratory Notes

**Published:** 1889-11

**Authors:** H. M. Whelpley


### Microscopical Laboratory Notes.

BY PROF. H. M. WHELPLEY, PH. G. F R. M. S., IN PHARMACEUTICAL ERA.

Cold Weather.—Do not permit the mounts to be reduced to the freezing temperature. Even those preparations in liquids that do not congeal at 32° F. will be injured by sudden or great changes in temperature.

Benzol is not benzin, and microscopists should remember it, even if some wholesale druggists do endeaver to sell benzin for benzol. I have seen work spoiled and time and patience lost by those who tried to use benzin for benzol.

Handling Thin Animal Sections.—Those who are accustomed to handling vegetable sections must remember when they come to work with animal specimens that the latter are much more liable to break or tear than vegetable sections. If this is not borne in mind valuable specimens will be ruined while handling them.

Another Use for Benzol.—This liquid is a great solvent of oils and grease. It will clean off the old grease that has been used to lubricate a joint, and leave the surface bright and clean for a fresh application of the lubricant. Benzol is very convenient for cleaning the spindle to a turntable when the table does not run smoothly.

Spoiled mounts are of no value, but the slides and cover glasses are. When a mount spoils beyond repair, place it in a wide-mouthed bottle containing equal parts of alcohol, oil of turpentine, coal oil and benzol. After a few days' maceration in this liquid, the slides and cover glasses may be wiped clean, and are then just as good as new.

To Handle Small Thin Sections.—A very convenient trowel for this purpose is made by inserting the head of a large needle in a penholder or other suitable handle; then filing the needle flat on two opposite sides, and breaking off the point. Such an instrument does not take up as much fluid with a small specimen as the ordinary trowel does. It also has other advantages over the customary methods of handling small thin sections of either animal or vegetable tissue.

The Astley-Cooper prize, amounting to $1,500, will be awarded in 1892. The question proposed is, "The Influence of Micro-organisms upon Inflammation." The papers must be written in English, or accompanied by an English translation, and should be addressed before January 1, 1892, to Guy's Hospital, London. The prize will not be awarded to two or three working conjointly.

